# Supplementary material for: Time series cross-correlation between home range and number of infected people during the COVID-19 pandemic in a suburban city
Source: PLoS One. 2022 Sep 1;17(9):e0267335. doi: 10.1371/journal.pone.0267335 (PMC9436136; doi:10.1371/journal.pone.0267335)
Supplement: Attachment — Submitted filename: Response to Reviewers.pdf [file pone.0267335.s001.pdf]

PONE-D-22-09773

Dear Reviewers and Academic Editor

Haruka KATO
Atsushi Takizawa

We appreciate the time and effort you have dedicated to providing insightful feedback on strengthening our manuscript. Thus, it is with great pleasure that we re-submit my article for further consideration. We have incorporated changes that reflect the detailed suggestions you have graciously provided. We also hope that our edits and the responses we provide below satisfy the issue and concern you have noted. The revised manuscript is marked up in red font with tracked change.

General Comment (Reviewer #1)

This study titled "Time series cross-correlation between home range and number of infected people during the medium term of COVID-19 Pandemic in a suburban city", is an interesting study where the author attempt to investigate the link between home range and COVID-19 infections. My comments are as below:

Author Response:

We appreciate your evaluation and meaningful comments on our manuscripts. According to your comments, we revised the manuscript as follows:

Comment No.1 (Reviewer #1)

1) There are a lot of studies published on this topic in recent times, the author needs to explain how this study is different from others.

Author Response:

We appreciate your meaningful suggestions regarding the novelty of this manuscript. Many studies analyzed the factors associated with the number of people infected with SARS-CoV-2. For example, it was reported that the risk of infections was correlated with chronic exposure to various outdoor air pollution [14-17]. Compared to those studies, the originality of this study is to focus on the human mobility to estimate the number of people infected with SARS-CoV-2. Besides, compared to studies the human mobility, the originality of this study is to clarify the time series cross-correlation between human mobility and the number of people infected with SARS-CoV-2.

Regarding the time series cross-correlation, it was found that the restriction of human mobility has a time lag effect on the growth rates of COVID-19 cases [25]. In China, human mobility is strongly correlated with COVID-19 cases, with lags of 10 days and a correlation coefficient of 0.68 from Janualy 17 to February 19, 2020 [26]. In Indonesia, human mobility is also correlated with COVID-19 cases with lags of 7-14 days and a correlation coefficient of about 0.50 from March 1 to July 31, 2020 [27]. Those studies indicate that the number of infected people correlated with human mobility

during the early stages of the pandemic. The novelty of this study is to analyze the correlation during the pandemic, which is from every Wednesday from April 2020 to July 2021.

The novelty was unclear in the previous manuscript. Therefore, we revised the explanation of novelty with some references. According to the additional references, the number of references is changed.

Manuscript Revision:

Ln 82-88	Many studies analyzed the factors associated with the number of people infected with SARS-CoV-2. For example, it was reported that the risk of infections was correlated with chronic exposure to various outdoor air pollution [14]. One effective policy for redusing the airpolusion is to reduce the human mobility related to the transportation infrastructure [15, 16]. During the lockdown restricting human mobility, it was found to reduce air pollution in some cities [17, 18]. Therefore, the originality of this study is to focus on human mobility to estimate the number of people infected with SARS-CoV-2. For the analysis of human mobility, many studies used mobile phone data. Regarding mobile phone data, GPS location history data is the state-of-the-art technology that has started to gain attention in the COVID-19 pandemic.
Ln 100-106	Compared to these studies, the originality of this study is to clarify the time series cross-correlation between human mobility and the number of people infected with SARS-CoV-2.<br>Regarding the time series cross-correlation, it was found that the restriction of human mobility has a time lag effect on the growth rates of COVID-19 cases [25]. In China, human mobility is strongly correlated with COVID-19 cases, with lags of 10 days and a correlation coefficient of 0.68 from January 17 to February 19, 2020 [26]. In Indonesia, human mobility is also correlated with COVID-19 cases with lags of 7-14 days and a correlation coefficient of about 0.50 from March 1 to July 31, 2020 [27].
Ln 97-100	Those studies indicate that the number of infected people correlated with human mobility during the early stages of the pandemic.<br>The novelty of this study is to analyze the time series cross-correlation during the pandemic, which is from every Wednesday from April 2020 to July 2021.
Ln 349-362	14. Marquès M, Domingo JL. Positive association between outdoor air pollution and the incidence and severity of COVID-19. A review of the recent scientific evidences. Environ Res. 2022;203: 111930. doi:10.1016/j.envres.2021.111930<br>15. Ali G, Abbas S, Qamer FM, Irteza SM. Environmental spatial heterogeneity of the impacts of COVID-19 on the top-20 metropolitan cities of Asia-Pacific. Sci Rep. 2021;11: 20339. doi:10.1038/s41598-021-99546-9<br>16. Abbas S, Ali G, Qamer FM, Irteza SM. Associations of air pollution concentrations

	and energy production dynamics in Pakistan during lockdown. Environ Sci Pollut Res. 2022;29: 35036–35047. doi:10.1007/s11356-021-18071-4
	17. Ali G, Abbas S, Qamer FM, Wong MS, Rasul G, Irteza SM, et al. Environmental impacts of shifts in energy, emissions, and urban heat island during the COVID-19 lockdown across Pakistan. J Clean Prod. 2021;291: 125806. doi:10.1016/j.jclepro.2021.125806
	18. 1. Ju MJ, Oh J, Choi Y-H. Changes in air pollution levels after COVID-19 outbreak in Korea. Sci Total Environ. 2021;750: 141521. doi:10.1016/j.scitotenv.2020.141521.
Ln 380-388	25. Wang S, Liu Y, Hu T. Examining the Change of Human Mobility Adherent to Social Restriction Policies and Its Effect on COVID-19 Cases in Australia. Int J Environ Res Public Health. 2020;17: 7930. doi:10.3390/ijerph17217930
	26. Xi W, Pei T, Liu Q, Song C, Liu Y, Chen X, et al. Quantifying the Time-Lag Effects of Human Mobility on the COVID-19 Transmission: A Multi-City Study in China. IEEE Access. 2020;8: 216752–216761. doi:10.1109/ACCESS.2020.3038995
	27. 1. Wijayanto AW, Wulansari IY. Human Mobility Patterns and Its Cross-Correlation with the COVID-19 Transmission in Jakarta, Indonesia. J Phys Conf Ser. 2021;1863: 012017. doi:10.1088/1742-6596/1863/1/012017

Comment No.2 (Reviewer #1)

2) Discussion section should be in more detail, and the author needs to compare the findings with other studies.

Author Response:

We appreciate your helpful suggestions regarding the discussion. The finding of this study is to clarify a slight positive correlation between home range and the number of infected people after one week during the COVID-19 pandemic in Ibaraki City. Especially, with regard to HR-length (Total Travel Distance), the CCC was found to be 0.4030 even at a lag level of six weeks, which had the most significant coefficient. The result was different from that of previous studies which cralified the strong coefficient during the early stage of the pandemic [25-27]. That result was also found that the COVID-19 cases were slightly compensated for positive indirect effects though human mobility [41]. the novelty of the present study is that a decrease in the home range is a weak factor correlated with a reduction in the number of infected people.

The findings was unclear in the previous manuscript. Therefore, we revised the explanation of discussion with some new references. The revision made easy to understand the importance of this study.

Manuscript Revision:

Ln 271-255	In conclusion, this study clarifies a slight positive correlation between home range and

	the number of infected people after one week during the COVID-19 pandemic in Ibaraki City. The positive correlations validated the results of previous studies [24,28]. In addition, prior research that analyzed the early stage period clarified a strong correlation [23,29]. The result of this study was important because it clarifies the slight positive correlation between home range and the number of infected people in the COVID-19 pandemic.
Ln 280-281	The result differed from previous studies that clarified the strong coefficient during the early stage of the pandemic [25-27]. Besides,
Ln 286-287	That result was also found that the COVID-19 cases were slightly compensated for positive indirect effects through human mobility [41]. The novelty of the present study is that a decrease in the home range is a weak factor correlated with a reduction in the number of infected people.
Ln 380-388	25. Wang S, Liu Y, Hu T. Examining the Change of Human Mobility Adherent to Social Restriction Policies and Its Effect on COVID-19 Cases in Australia. Int J Environ Res Public Health. 2020;17: 7930. doi:10.3390/ijerph17217930<br>26. Xi W, Pei T, Liu Q, Song C, Liu Y, Chen X, et al. Quantifying the Time-Lag Effects of Human Mobility on the COVID-19 Transmission: A Multi-City Study in China. IEEE Access. 2020;8: 216752–216761. doi:10.1109/ACCESS.2020.3038995<br>27. 1. Wijayanto AW, Wulansari IY. Human Mobility Patterns and Its Cross-Correlation with the COVID-19 Transmission in Jakarta, Indonesia. J Phys Conf Ser. 2021;1863: 012017. doi:10.1088/1742-6596/1863/1/012017
Ln 421-422	41.Damette O, Mathonnat C, Goutte S. Meteorological factors against COVID-19 and the role of human mobility. Troccoli A, editor. PLoS One. 2021;16: e0252405. doi:10.1371/journal.pone.0252405

General Comment (Reviewer #2)

This manuscript unveils the time series cross-correlation between home range and number of infected people during the medium-term of the COVID-19 pandemic in a suburban city. The article is in good shape and could be accepted for possible publication after addressing the comments given below:

Author Response:

We appreciate your meaningful suggestions regarding each chapters and sections. Your comments are detail and esy to understand. The details of the revision are as follows.

Comment No.1 (Reviewer #2)

Title:
The "medium-term" may not be suitable in the title, which may put reader to guess about the term.

Author Response:

We appreciate your suggestions regarding the title. What we want to explain is not early stage of pandemic but several waves of the pandemic. However, as your suggestion, the term of "Medium-term" was unclear. Therefore, this study set the pandemic period as from January 2020 to July 2021. That is because vaccination progress after July 2021 in Japan. We revised the explanation of pandemic period to correct to avoid misunderstanding.

Manuscript Revision:

Ln 1-2	Time series cross-correlation between home range and number of infected people during the COVID-19 pandemic in a suburban city
Ln 19-20	This study aims to clarify the correlation between home range and the number of people infected with SARS-CoV-2 during the COVID-19 pandemic in Ibaraki City.
Ln 22-24	Results reveal a slight positive correlation between home range and the number of infected people after one week during the COVID-19 pandemic.
Ln 41-42	Does the control of home range correlate with a reduction in the number of infected people during the COVID-19 pandemic?
Ln 52-53	This study aims to clarify the time series cross-correlation between home range and the number of people infected with SARS-CoV-2 during the COVID-19 pandemic in a suburban city.
Ln 56-60	In this study, the pandemic period was set from January 2020 to July 2021. In Japan, the first case of SARS-CoV-2 infection was confirmed in January 2020. From April to May 2020, the first state of emergency was declared for all prefectures [14]. In 2021, states of emergency were declared repeatedly, mainly in Osaka prefectures, from January to February, April to June. After July 2021, vaccination progress in Japan. Therefore, the analysis period, from January 2020 to July 2021, is thought to be the COVID-19 pandemic period in Japan.

Ln 99-100	The novelty of this study is to analyze the correlation during the pandemic, which is from every Wednesday from April 2020 to July 2021.
Ln 271-272	In conclusion, this study clarifies a slight positive correlation between home range and the number of infected people after one week during the COVID-19 pandemic in Ibaraki City.
Ln 274-276	The result of this study was significant because it clarifies the slight positive correlation between home range and the number of infected people in the COVID-19 pandemic.

Comment No.2 (Reviewer #2)

Abstract: Could have been written better. Also include and elaborate the approach. The first sentence should be broken into 2-3 clear sentences. SBM – write in full when appeared first in the abstract and introduction.

Author Response:

We appreciate your helpful suggestions regarding the abstract. As your suggestion, we added the approach with three sentences, including the research question. This revision made essy to understand the importance of this study.

Manuscript Revision:

Ln 16-19	However, the imposition of emergency restrictions had significant negative impacts on citizens' daily lives. As vaccination progresses, we need to consider more effective measures to control the spread of the infection. The research question of this study is as follows: Does the control of home range correlate with a reduction in the number of infected people during the COVID-19 pandemic?

Comment No.3 (Reviewer #2)

Introduction:

The introduction is very good; the authors demonstrate a thorough knowledge of the published literature and highlight the importance and background to carry out this investigation. Generally, it is reasonable, to follow the current pattern, which is more clear, but it might be good to follow the stat of the art format for the introduction. Then, elaborate the objectives with focus before jumping on the description of sessions.

Also include some regional and local studies on the impact of covid-19 on mobility and its associated impact other environmental indicators for instance air pollution, it has been noted the different mobility patterns and measures across the Asia. Some references are given below which could be considered.

Figure 1 should be move in the Location History Data.

Figure 1 – Please add a proper study area map with location of Japan and the study area in index maps.

"Changes in air pollution levels after COVID-19 outbreak in Korea." Science of the Total Environment 750 (2021): 141521.

Associations of air pollution concentrations and energy production dynamics in Pakistan during lockdown. Environ Sci Pollut Res (2022). https://doi.org/10.1007/s11356-021-18071-4

Environmental Impacts of Shifts in Energy, Emissions, and Urban Heat Island during the COVID-19 Lockdown Across Pakistan. J. Clean. Prod. 125806. https://doi.org/10.1016/j.jclepro.2021.125806

"Positive association between outdoor air pollution and the incidence and severity of COVID-19. A review of the recent scientific evidences." Environmental Research (2021): 111930.

Environmental Spatial Heterogeneity of the Impacts of COVI-19 on the Top-20 Metropolitan Cities of Asia-Pacific. Nature Scientific Reports, 11, 20339. 2021

Author Response:

We appreciate your evaluation and helpful suggestions regarding the introduction. We revised the purpose of this study more detail. Besides, the originality and novelty was unclear in the previous manuscrtipt. The originality of this study is to clarify the time series cross-correlation between human mobility and the number of people infected with SARS-CoV-2. Besides, the novelty of this study is to analyze the correlation during the pandemic, which is from every Wednesday from April 2020 to July 2021. We revised the explanation with some references which noted the different mobility patterns and measures across the Asia. The revision made easy to understand.

Manuscript Revision:

Ln 56-60	In this study, the pandemic period was set from January 2020 to July 2021. In Japan, the first case of SARS-CoV-2 infection was confirmed in January 2020. From April to May 2020, the first state of emergency was declared for all prefectures [14]. In 2021, states of emergency were declared repeatedly, mainly in Osaka prefectures, from January to February, April to June. After July 2021, vaccination progress in Japan. Therefore, the analysis period, from January 2020 to July 2021, is thought to be the COVID-19 pandemic period in Japan. A
Ln 82-88	Many studies analyzed the factors associated with the number of people infected with SARS-CoV-2. For example, it was reported that the risk of infections was correlated with chronic exposure to various outdoor air pollution [14]. One effective policy for redusing the airpolusion is to reduce the human mobility related to the transportation infrastructure [15, 16]. During the lockdown restricting human mobility, it was found to reduce air pollution in some cities [17, 18]. Therefore, the originality of this study is to focus on human mobility to estimate the number of people infected with SARS-CoV-2. For the analysis of human mobility, many studies used mobile phone data. Regarding mobile phone data, GPS location history data is the state-of-the-art technology that has started to gain attention in the COVID-19 pandemic.

Ln 100-106	Compared to these studies, the originality of this study is to clarify the time series cross-correlation between human mobility and the number of people infected with SARS-CoV-2.<br>Regarding the time series cross-correlation, it was found that the restriction of human mobility has a time lag effect on the growth rates of COVID-19 cases [25]. In China, human mobility is strongly correlated with COVID-19 cases, with lags of 10 days and a correlation coefficient of 0.68 from January 17 to February 19, 2020 [26]. In Indonesia, human mobility is also correlated with COVID-19 cases with lags of 7-14 days and a correlation coefficient of about 0.50 from March 1 to July 31, 2020 [27].
Ln 113-118	Those studies indicate that the number of infected people correlated with human mobility during the early stages of the pandemic.<br>The novelty of this study is to analyze the time series criss-correlation during the pandemic, which is from every Wednesday from April 2020 to July 2021.
Ln 349-362	14. Marquès M, Domingo JL. Positive association between outdoor air pollution and the incidence and severity of COVID-19. A review of the recent scientific evidences. Environ Res. 2022;203: 111930. doi:10.1016/j.envres.2021.111930<br>15. Ali G, Abbas S, Qamer FM, Irteza SM. Environmental spatial heterogeneity of the impacts of COVID-19 on the top-20 metropolitan cities of Asia-Pacific. Sci Rep. 2021;11: 20339. doi:10.1038/s41598-021-99546-9<br>16. Abbas S, Ali G, Qamer FM, Irteza SM. Associations of air pollution concentrations and energy production dynamics in Pakistan during lockdown. Environ Sci Pollut Res. 2022;29: 35036–35047. doi:10.1007/s11356-021-18071-4<br>17. Ali G, Abbas S, Qamer FM, Wong MS, Rasul G, Irteza SM, et al. Environmental impacts of shifts in energy, emissions, and urban heat island during the COVID-19 lockdown across Pakistan. J Clean Prod. 2021;291: 125806. doi:10.1016/j.jclepro.2021.125806<br>18. 1. Ju MJ, Oh J, Choi Y-H. Changes in air pollution levels after COVID-19 outbreak in Korea. Sci Total Environ. 2021;750: 141521. doi:10.1016/j.scitotenv.2020.141521.
Ln 380-388	25. Wang S, Liu Y, Hu T. Examining the Change of Human Mobility Adherent to Social Restriction Policies and Its Effect on COVID-19 Cases in Australia. Int J Environ Res Public Health. 2020;17: 7930. doi:10.3390/ijerph17217930<br>26. Xi W, Pei T, Liu Q, Song C, Liu Y, Chen X, et al. Quantifying the Time-Lag Effects of Human Mobility on the COVID-19 Transmission: A Multi-City Study in China. IEEE Access. 2020;8: 216752–216761. doi:10.1109/ACCESS.2020.3038995<br>27. 1. Wijayanto AW, Wulansari IY. Human Mobility Patterns and Its Cross-Correlation with the COVID-19 Transmission in Jakarta, Indonesia. J Phys Conf Ser. 2021;1863:

	012017. doi:10.1088/1742-6596/1863/1/012017

Comment No.4 (Reviewer #2)

Materials and Methods<br>Methods are technically strong and well explained.<br>Figures' captions are good and self-explanatory.

Author Response:

We appreciate your evaluation on the Material and Methods Section.

Comment No.5 (Reviewer #2)

Results:<br>Results are described in detail.<br>Line 177 Section 3.1 in not mentioned in the manuscript – probable formatting issues.<br>Figures' captions are good and self-explanatory.

Author Response:

We appreciate your evaluation on Result Section. This section removed the word of " *In Section 3.1*".

Manuscript Revision:

Ln 207-209	Fig 3 shows the box-plot diagrams of weekly changes in HR-length (Farthest Distance) and HR-length (Total Travel Distance) from April 2020 to July 2021; Fig 4 shows the bar-graphs of monthly changes in HR-length (Farthest Distance) and HR-length (Total Travel Distance) from April 2020 to July 2021.

Comment No.6 (Reviewer #2)

Discussion<br>Authors have combined Discussion with Conclusion which is not a usual practice but could be acceptable. It would be good to add relevance and prospective of the current study for wider area coverage using existing and upcoming biomass measurement missions.

Author Response:

We appreciate your helpful suggestions regarding the Discussion Chapter. Regarding your comments, it is important to study the other factors that correlate with the number of infected cases, such as air pollution using existing and upcoming biomass measurement missions for the urban sustainability. Therefore, we added the explanation as the future research.

Manuscript Revision:

Ln 312-313	In addition, for urban sustainability, it is essential to study the other factors that correlate with the number of infected cases, such as air pollution, using existing and upcoming biomass measurement missions.

Comment No.7 (Reviewer #2)

Conclusion<br>No Comments.

Author Response:

We appreciate your evaluation on the Conclusion Section.

General Comment (Academic Editor)

When submitting your revision, we need you to address these additional requirements.

Author Response:

We appreciate your technical suggestions regarding manuscript style, informed consent, COI, data availability, and CC BY 4.0 license. We response to those five points. The revisions were as follows:

Comment No.1(Academic Editor)

1. Please ensure that your manuscript meets PLOS ONE's style requirements, including those for file naming. The PLOS ONE style templates can be found at https://journals.plos.org/plosone/s/file?id=wjVg/PLOSOne_formatting_sample_main_body.pdf and https://journals.plos.org/plosone/s/file?id=ba62/PLOSOne_formatting_sample_title_authors_affiliations.pdf

Author Response:

We appreciate your comments regarding the format of the manuscript. We revised the manuscript to meet the PLOS ONE's style requirements. The revisions were all of the manuscript. Therefore, we want you to check the revised manuscript.

Comment No.2(Academic Editor)

2. Please provide additional details regarding participant consent. In the ethics statement in the Methods and online submission information, please ensure that you have specified (1) whether consent was informed and (2) what type you obtained (for instance, written or verbal, and if verbal, how it was documented and witnessed). If your study included minors, state whether you obtained consent from parents or guardians. If the need for consent was waived by the ethics committee, please include this information.

If you are reporting a retrospective study of medical records or archived samples, please ensure that you have discussed whether all data were fully anonymized before you accessed them and/or whether the IRB or ethics committee waived the requirement for informed consent. If patients provided informed written consent to have data from their medical records used in research, please include this information.

Author Response:

We appreciate your suggestions regarding the informed consent. This study used the LH data, which obtaining consent from users, who contracted with a specific mobile phone carrier company or installed specific applications. All participants were provided with information regarding the type of data collected, purpose of use and provision to third parties, and a privacy policy. That means that the informed consent was obtained from all subjects based on the privacy policy. However, the consent is not written paper because it is digital data using mobile phone. Additionally, the subjects can stop sending their human mobility data at any time by changing their mobile phones' settings. This research

protocol was approved by the ethics committee of the Graduate School of Life Science, Osaka City University (No.21-40). We added the explanation on informed consent of the data.

Manuscript Revision:

Ln 129-131	That means that informed consent was obtained from all subjects based on the privacy policy. However, the consent is not written paper because it is digital data using mobile phone. Additionally, the subjects can stop sending their human mobility data anytime by changing their mobile phones' settings.

Comment No.3(Academic Editor)

3. Thank you for stating the following in your Competing Interests section:

"NO"

Please complete your Competing Interests on the online submission form to state any Competing Interests. If you have no competing interests, please state "The authors have declared that no competing interests exist.", as detailed online in our guide for authors at http://journals.plos.org/plosone/s/submit-now

This information should be included in your cover letter; we will change the online submission form on your behalf.

Author Response:

We appreciate suggestions regarding the competing interests. In the coverlette, we wrote the "There are no conflicts of interest to declare". However, the explanation was unclrear. As your comments, the authors have declared that no competing interests exist. Therefore, we revised the sentence regarding the competing interests in the system and coverletter.

Manuscript Revision:

Cover Letter	the authors have declared that no competing interests exist

Comment No.4(Academic Editor)

4. In your Data Availability statement, you have not specified where the minimal data set underlying the results described in your manuscript can be found. PLOS defines a study's minimal data set as the underlying data used to reach the conclusions drawn in the manuscript and any additional data required to replicate the reported study findings in their entirety. All PLOS journals require that the minimal data set be made fully available. For more information about our data policy, please see http://journals.plos.org/plosone/s/data-availability.

Upon re-submitting your revised manuscript, please upload your study's minimal underlying data set as either Supporting Information files or to a stable, public repository and include the relevant URLs, DOIs, or accession numbers within your revised cover letter. For a list of acceptable repositories, please see http://journals.plos.org/plosone/s/data-availability#loc-recommended-repositories. Any potentially

identifying patient information must be fully anonymized.<br>Important: If there are ethical or legal restrictions to sharing your data publicly, please explain these restrictions in detail. Please see our guidelines for more information on what we consider unacceptable restrictions to publicly sharing data: http://journals.plos.org/plosone/s/data-availability#loc-unacceptable-data-access-restrictions. Note that it is not acceptable for the authors to be the sole named individuals responsible for ensuring data access.<br>We will update your Data Availability statement to reflect the information you provide in your cover letter.

Author Response:

We appreciate you taking the time to point this out to me regarding the data availability statements. The data of the number of people infected with SARS-CoV-2 is available from references [40], which is website of Ibaraki City Government. On the other hand, the LH data cannot be shared publicly because of mobile phone users' confidentiality and legal restrictions with authors and Agoop corporation. However, researchers can used the LH data, if they contract with the Agoop Corpolation Institutional Data Access (contact via ESRI Japan) for researchers who meet the criteria for access to confidential data. We added the explanation in the systems and cover letter.

Manuscript Revision:

Cover Letter / System	Regarding the data availability statement, the data of the number of people infected with SARS-CoV-2 is available from references of website of Ibaraki City Government. The link of the website is the following (in Japanese): https://www.city.ibaraki.osaka.jp/kikou/kenkoi/kenkou/menu/2covid19.html.<br>On the other hand, the GPS Location History data (LH data) cannot be shared publicly because of mobile phone users' confidentiality and the legal restrictions with authors and Agoop corporation. However, researchers can use the LH data, if they contract with the Agoop Corpolation Institutional Data Access (contact via ESRI Japan) for researchers who meet the criteria for access to confidential data. The contact information link is the following E-mail address: gisinfo@esrij.com (ESRI Japan, Customer Support Service)

Comment No.5(Academic Editor)

5. We note that Figures 1 and 2 in your submission contain map images which may be copyrighted. All PLOS content is published under the Creative Commons Attribution License (CC BY 4.0), which means that the manuscript, images, and Supporting Information files will be freely available online, and any third party is permitted to access, download, copy, distribute, and use these materials in any way, even commercially, with proper attribution. For these reasons, we cannot publish previously copyrighted maps or satellite images created using proprietary data, such as Google software (Google Maps, Street View, and Earth). For more information, see our copyright guidelines:

http://journals.plos.org/plosone/s/licenses-and-copyright.
We require you to either (1) present written permission from the copyright holder to publish these figures specifically under the CC BY 4.0 license, or (2) remove the figures from your submission:

a. You may seek permission from the original copyright holder of to Figures 1 and 2 publish the content specifically under the CC BY 4.0 license.

We recommend that you contact the original copyright holder with the Content Permission Form (http://journals.plos.org/plosone/s/file?id=7c09/content-permission-form.pdf) and the following text:
"I request permission for the open-access journal PLOS ONE to publish XXX under the Creative Commons Attribution License (CCAL) CC BY 4.0 (http://creativecommons.org/licenses/by/4.0/). Please be aware that this license allows unrestricted use and distribution, even commercially, by third parties. Please reply and provide explicit written permission to publish XXX under a CC BY license and complete the attached form."
Please upload the completed Content Permission Form or other proof of granted permissions as an "Other" file with your submission.
In the figure caption of the copyrighted figure, please include the following text: "Reprinted from [ref] under a CC BY license, with permission from [name of publisher], original copyright [original copyright year]."

b. If you are unable to obtain permission from the original copyright holder to publish these figures under the CC BY 4.0 license or if the copyright holder's requirements are incompatible with the CC BY 4.0 license, please either i) remove the figure or ii) supply a replacement figure that complies with the CC BY 4.0 license. Please check copyright information on all replacement figures and update the figure caption with source information. If applicable, please specify in the figure caption text when a figure is similar but not identical to the original image and is therefore for illustrative purposes only.

The following resources for replacing copyrighted map figures may be helpful:
USGS National Map Viewer (public domain): http://viewer.nationalmap.gov/viewer/
The Gateway to Astronaut Photography of Earth (public domain): http://eol.jsc.nasa.gov/sseop/clickmap/
Maps at the CIA (public domain): https://www.cia.gov/library/publications/the-world-factbook/index.html and https://www.cia.gov/library/publications/cia-maps-publications/index.html
NASA Earth Observatory (public domain): http://earthobservatory.nasa.gov/
Landsat: http://landsat.visibleearth.nasa.gov/
USGS EROS (Earth Resources Observatory and Science (EROS) Center) (public domain): http://eros.usgs.gov/#
Natural Earth (public domain): http://www.naturalearthdata.com/

Please review your reference list to ensure that it is complete and correct. If you have cited papers that

have been retracted, please include the rationale for doing so in the manuscript text, or remove these references and replace them with relevant current references. Any changes to the reference list should be mentioned in the rebuttal letter that accompanies your revised manuscript. If you need to cite a retracted article, indicate the article's retracted status in the References list and also include a citation and full reference for the retraction notice.

Author Response:

We appreciate your helpful suggestions regarding the figure copyright. The map in figure was made using the Arc GIS PRO, not the Google Map and Earth. Therefore, we contacted with the ESRI Japan, which service provider of Arc GIS PRO in Japan. In the Arc GIS PRO, the satellite map was open source in Japan. Therefore, we can use the satellite map in Fig 1-2 without any content permissions, in compliance with the copyright of ESRI REST Services Directory. That means the permission granted by ESRI Japan includes permission to publish content under CC BY 4.0. Therefore, we added the explanation and reference in the manuscript.

Manuscript Revision:

Ln 66	The map in Fig 1 was the open-source Arc GIS PRO and complied with the copyright [10].
Ln 174-175	The map in Fig 2 was the open-source Arc GIS PRO and complied with the copyright [10].
Ln 339-340	10. ArcGIS REST Services Directory [cited 2020 June 29]. Available from: https://services.arcgisonline.com/ArcGIS/rest/services/World_Imagery/MapServer

Again, thank you for giving us the opportunity to strengthen our manuscript with your valuable comments. We have worked hard to incorporate your feedback and hope that these revisions persuade you to accept our submission.

Sincerely,
